# Clinical outcomes of participants of a TB prevalence survey with an abnormal chest X-ray but no evidence of TB disease after a median follow-up of 9 months in Zambia and South Africa

**DOI:** 10.1371/journal.pgph.0003787

**Published:** 2025-06-20

**Authors:** Maria Ruperez, Jacob Busang, Linda Mureithi, Kwame Shanaube, Eveline Klinkenberg, Thomas Gachie, James M. Burnett, Barry Kosloff, Petra de Haas, Richard Hayes, Sarah Fidler, Ab Schaap, Sian Floyd, Helen Ayles, on behalf of TREATS study team

**Affiliations:** 1 Clinical Research Department, London School of Hygiene & Tropical Medicine (LSHTM), London, United Kingdom; 2 Health Systems Trust, Cape Town, South Africa; 3 Africa Health Research Institute, Mtubatuba, KwaZulu-Natal, South Africa; 4 Zambart, University of Zambia School of Medicine, Lusaka, Zambia; 5 Department of Global Health, Amsterdam University Medical Centers, Amsterdam, The Netherlands; 6 Consultant, ConnectTB, The Hague, The Netherlands; 7 KNCV Tuberculosis Foundation, The Hague, The Netherlands; 8 Department of Infectious Disease Epidemiology and International Health, London School of Hygiene & Tropical Medicine (LSHTM), London, United Kingdom; 9 Imperial College London, London, United Kingdom; University College of London, UNITED KINGDOM OF GREAT BRITAIN AND NORTHERN IRELAND

## Abstract

WHO recommends computer-aided detection (CAD) in chest X-ray (CXR) for systematic screening of TB. Increased detection of individuals with high CAD score but without bacteriologically confirmed TB can be expected, requiring guidance on their clinical management. We followed participants of a TB prevalence survey (TBPS) in Zambia and South Africa with a high CAD score but no bacteriologically confirmed TB over a median time of 9 months and assessed their clinical outcomes. At the TBPS participants with TB-suggestive symptoms or a CAD score ≥40 submitted two sputum samples for Xpert-Ultra testing, and, an additional sample was collected the next day for liquid culture and Xpert-ultra testing. Participants with a CAD score ≥70 and no bacteriologically confirmed TB were eligible for follow-up. At follow-up visit participants were asked about TB symptoms and treatment, underwent a repeat CXR with CAD, and those with either TB-suggestive symptoms or a CAD score ≥70 at follow-up submitted a sputum sample for Xpert-Ultra testing. A composite “clinical” outcome was defined based on changes in CAD-score and TB-suggestive symptoms between the TBPS and the follow-up. Of the 254 eligible TBPS participants 162 (65%) completed follow-up. Most of the participants self-reported previous TB (65% 105/162), were from Zambia (79%, 128/162,) and male (70%, 97/162). Overall, 43% (70/162) participants progressed clinically/remained radiologically abnormal and 6% (10/162) developed TB between the TBPS and the follow-up, with an overall TB incidence rate of 7% per year (95% CI: 3.8-13.3). Patients with high CAD score but no bacteriological confirmation may have had a past TB or other pulmonary lesions identified in the CXR, which may need to be investigated. Also, these participants may be at risk of progressing to TB over time and could benefit from a follow-up visit and from repeated assessment of symptoms and CXR.

## 1. Introduction

Despite being a curable and preventable disease, tuberculosis (TB) is still one of the leading causes of death globally. In 2023, an estimated 10.8 million people fell ill with tuberculosis and 1.25 million people died [1]. Ending the TB epidemic by 2030 is among the United Nations Sustainable Development Goals, but achieving this ambitious target requires identification of all people with TB and ensuring early diagnosis and initiation of care [2]. One of the key strategies that has been proposed for this purpose is systematic screening for TB disease among the general population in areas with an estimated TB prevalence of 0.5% or higher [3].

Symptom screening has shown to have important limitations for detecting TB. It has low and variable sensitivity depending on the prevalence of other non-TB conditions and is subjective on the interpretation of the provider conducting the screen and the person being screened. Renewed interest in Chest X-Ray (CXR) as a screening tool for TB in low and middle-income-countries (LMIC) with high TB burden has followed findings from various recent TB prevalence surveys (TBPS) showing that CXR has high sensitivity to detect pulmonary TB [3,4]. New innovative tools, such as the use of artificial-intelligence-based computer-aided detection (CAD) of TB on digital radiographs, have been developed enabling TB diagnosis in places with limited availability of radiologists or expert clinicians. Several commercial CAD products are now available, with algorithms that score CXRs on a scale from 0 to 100 according to the probability of TB-associated abnormalities.

In 2021, WHO issued a new recommendation within its tuberculosis screening guidelines: the approval of CAD to analyse CXRs for tuberculosis detection in place of human readers. This was based on evidence suggesting that the accuracy of CAD approximates that of radiologists in identifying tuberculosis on CXRs [3,5,6].

However, CAD has insufficiently high specificity for it to be used on its own, as a diagnostic test for TB, and it requires a bacteriological confirmatory test among individuals with high scores. Having an abnormal CXR (macroscopic pathology) without bacteriologically confirmed TB (no infectiousness) is a common finding in TBPS, though the clinical meaning and the appropriate management of these individuals is unclear [7–9]. If CAD is widely implemented for TB screening, clear clinical pathways and guidance will be required for individuals who are identified by CAD as having a CXR that is “abnormal – likely TB” but in whom bacteriological tests do not identify pulmonary TB.

In this study, participants in a TBPS in 4 communities in Lusaka Province in Zambia and the Western Cape Province of South Africa (SA) who had high CAD scores but tested negative on bacteriological testing for *Mycobacterium tuberculosis* (Mtb), were assessed for clinical, radiological, and bacteriological outcomes at a follow-up visit, 5–21 months later.

## 2. Methods

### 2.1. Ethics statement

This study was approved by the London School of Hygiene and Tropical Medicine Ethics Committee and by the in-country regulatory boards, University of Zambia Biomedical Research Ethics Committee (UNZABREC) and Pharma-Ethics Ltd in South Africa. The study research assistant, trained for this purpose, sought informed consent from participants. Individuals gave written informed consent for study participation, and for individuals aged 15–17 years, assent and parental consent were obtained.

### 2.2. Study design and procedures

This was a prospective observational study nested within the Tuberculosis Reduction through Expanded Anti-retroviral Treatment and Screening (TREATS) TBPS aiming to evaluate changes in clinical and bacteriological outcomes over time in individuals who were not diagnosed with TB. The study was conducted in three communities in Zambia, located in the Lusaka district (Lusaka Province) and in one community in South Africa, located in the Metro district (Western Cape Province). These communities have high HIV and TB prevalence [10,11].

We defined “clinical” outcomes at the follow-up visit based on (a) a repeated CXR evaluation using CAD scores, and (b) reported TB suggestive symptoms. Bacteriological outcomes were based on Xpert MTB/RIF Ultra (Xpert-Ultra) testing for TB, using one sputum sample taken on-the-spot the day of the follow-up visit.

#### 2.2.1. TREATS-TBPS procedures.

**Fig 1:**
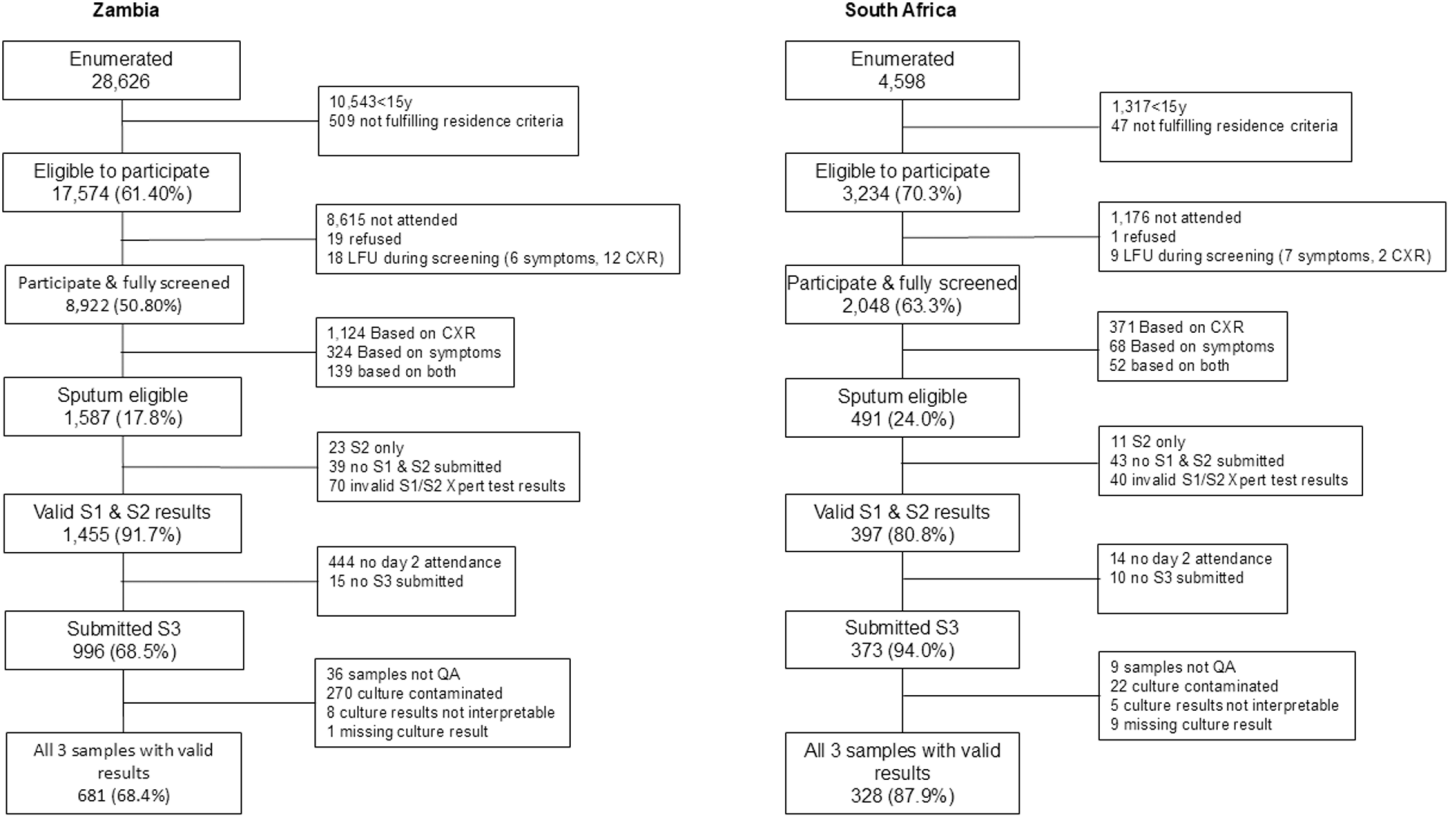
TREATS Intensive Diagnostic Phase (IDP) flow diagram.

The TREATS TBPS was conducted between 2019–2021, across 21 urban and peri-urban communities in Zambia and the Western Cape province of South Africa and aimed to evaluate the impact of the HPTN 071 - Population Effects of Antiretroviral Therapy to Reduce HIV Transmission (PopART) intervention on TB prevalence. The PopART intervention consisted of a population-level symptom screening for TB combined with universal testing and treatment for HIV in the community [11,12]. The TREATS TBPS consisted of two phases, an “intensive diagnostic phase” (IDP) conducted in the first 4 communities in which the current study was nested, and a non-intensive diagnostic phase (non-IDP) in the remaining 17 communities [13]. This study was nested within the IDP, which aimed to achieve a better understanding of alternative ways to screen for and diagnose TB [10]. See IDP flow diagram in Fig 1.

In the 4 IDP communities, the TBPS was conducted from February to October 2019 and follow-up of participants took place from 05^th^ February – 30^th^ March 2020 in the Zambian communities and from 28^th^ October 2020 – 14^th^ Jan 2021 in the South African community.

Detailed description of the TBPS study procedures in the IDP communities is presented elsewhere [10]. Briefly, all households in randomly selected areas of the study communities were enumerated (listed) and household members aged ≥15 years were invited to participate in the TBPS. Centrally, in each survey area, a OneStopTB Platform (Delft Imaging, the Netherlands) containing a digital X-ray and Xpert instrument was stationed. All eligible participants were asked about previous or current TB treatment, screened for symptoms suggestive of TB and had a digital CXR performed. The CXRs were read and scored using CAD reading software (CAD4TB version 5.0) that provided an output score between 0 and 100 related to the probability of the participant having TB. Individuals who were positive on the symptom screening (had cough≥ 2 weeks OR at least two symptoms among the following: (i) fever ≥ 2 weeks (ii) chest pain ≥ 2 weeks (iii) night sweats ≥ 2 weeks (iv) unexpected weight loss ≥4 weeks (v) cough of any duration) and/or had a CAD score ≥40 were considered eligible for sputum examination. Sputum eligible (SE) participants were asked to provide two “on-the-spot” sputum samples taken at least 30 minutes apart, which were tested using Xpert-Ultra in the OneStopTB platform on the same day. All individuals who were SE were requested to return the following day to receive the Xpert-Ultra results and for clinical management. As part of the IDP, all those reporting back on day 2 were also asked to provide a third “on-the-spot” sputum sample that was transported to a central laboratory for liquid culture and Xpert-Ultra testing. On this same day, the medical officer (MO) reviewed all available screening and test results and reached a clinical decision on referral for TB treatment or other health care.

Definitions of positive and negative results on Xpert-Ultra and culture in the TBPS are defined in detail elsewhere [10]. Briefly, Xpert results were defined as Xpert-positive if either of the 2 sputum samples was graded as MTB-detected-trace, very low or above, and as Xpert-negative if neither sputum sample was positive and at least one had a negative test result. The “final” culture result for each sputum sample that was collected for culture testing was defined based on the combination of outcomes from the two 2 mycobacteria growth indicator tubes (MGIT). The result was classified as “culture-positive” if ≥1 tube result was positive for MTB. Among those that were not “culture-positive” they were classified as “culture-negative” if ≥1 tube was negative for MTB or ≥1 tube was positive for Non-Tuberculous Mycobacteria (NTM), “contaminated” if both tubes were contaminated, and as “non-interpretable” if either both tubes were non-interpretable, or one was non-interpretable and one was contaminated. For this study a “valid” culture result was one that was “culture-positive” or “culture-negative” and from a batch where the positive control grew, and the negative control did not.

For this study, we considered that a participant had “bacteriologically confirmed TB” if at least one Xpert-Ultra or valid culture result was positive for MTB, and “no bacteriologically confirmed TB” if there were no positive test results and at least one of the Xpert-Ultra and valid culture results was negative. If they had no results for both Xpert tests performed on sputum samples submitted on the first day and for the culture performed on sputum submitted on day 2 they were excluded from the analysis. Having no results in the tests could be caused by not submitting sputum samples (participants not able to expectorate, refusing and/or not attending day 2) or from getting inconclusive results on the test. However, these participants were all assessed by clinical officer and linked to care if needed.

All participants were asked about HIV status and were offered HIV testing and counselling if they did not self-report that they were a person living with HIV (PLHIV). Participants who were newly diagnosed with TB (either clinical and bacteriologically confirmed TB) or with HIV were referred to the nearest health facility for treatment initiation and linkage to care, following the country’s national TB and HIV treatment guidelines.

#### 2.2.2. Follow-up procedures.

Participants in the TREATS-TBPS in the IDP sites who had a CAD score ≥ 70 on the CXR with “no bacteriologically confirmed TB” at the TBPS were eligible for follow-up. Eligible participants were followed up at their household and invited to participate in the follow-up visit. Following informed consent participants were administered a questionnaire on current TB symptoms, and whether they had started TB treatment since they participated in the TBPS. All were offered a digital CXR taken at a nearby site. In Zambia a “backpack” mobile X-ray (Delft Light, Delft Imaging, the Netherlands) was used whereas in South Africa CXRs were taken at the X-ray unit of the OneStopTB platform. The CXRs were read and scored by the CAD4TB software (CAD4TB version 5.0). Any participants who had symptoms suggestive of TB or a CAD score ≥70 in the follow-up survey were asked to submit one on-the-spot sputum sample that was sent to a central laboratory for testing with Xpert-Ultra (in South Africa samples were not collected for those who had started treatment since the TBPS).

### 2.3. Definition and classification of outcomes

#### 2.3.1. Radiological outcomes.

Among those eligible for the follow-up survey, we pragmatically categorised the participants‘baseline (at the time of the TBPS) CAD scores into three categories [70–79], [80–89], [90–100]. We then quantified the change in CAD scores as the difference between CAD score at follow-up and at baseline.

Radiological outcomes in this study were categorised as “improved,” “progressed or remained abnormal,” or “intermediate” based on CAD score changes between follow-up and baseline. An “improved” outcome was defined as a reduction of an absolute change of 10 or more in the CAD score at follow-up compared to baseline (e.g., reduction from a score of 80 to ≤70). Conversely, a “progressed or remained abnormal” radiological outcome was defined as a 10 or more increase in the CAD score at follow-up amongst those who had baseline CAD scores of 70–84. Individuals with baseline and follow-up CAD scores ≥85 were also included in the category of a “progressed or remained abnormal” outcome due to limited scope to increase by 10 or more. An “intermediate” outcome was assigned when there was no substantial change (≤10) in either direction (Table 1).

**Table 1:** Definition and classification of clinical, and radiological outcomes.

Clinical outcome	Radiological outcome		Symptoms at follow-up*
**Improved**	**Improved** *Participants who had a reduction in the CAD score of 10 or more in the follow-up from baseline CAD score*	**AND**	**Asymptomatic**
**Intermediate**	**Intermediate** *Participants with no change in CAD score or change not* *exceeding 10 in the follow-up from baseline CAD score*	**AND**	**Asymptomatic**
**Progressed clinically/remained radiologically abnormal**	**Progressed or remained abnormal** *Participants who had an increase of* ≥*10 in the CAD score at follow-up amongst those who had baseline CAD scores of 70–84* OR *Participants who had a baseline CAD score* ≥*85 and the CAD score at follow-up was also* ≥*85.*	**OR**	**Symptomatic**

*Having one of the following: Cough ≥2 weeks OR at least two symptoms among the following (i) fever ≥ 2 weeks (ii) chest pain ≥ 2 weeks (iii) night sweats ≥ 2 weeks (iv) unexpected weight loss ≥4 weeks (v) cough of any duration.

#### 2.3.2. Symptom outcomes.

We used the same TB symptom-screening criteria that were used to define sputum-eligibility in the TBPS to categorise individuals according to their TB symptoms at follow-up. Those who did not meet these symptom-screening criteria were classified as asymptomatic for TB at the follow-up (Table 1).

#### 2.3.3. Clinical outcomes.

We defined a composite “clinical” outcome combining the radiological and TB symptoms outcomes. Participants who had either a “progressed or remained abnormal” radiological outcome OR were symptomatic for TB at follow-up were defined as having “progressed clinically/remained radiologically abnormal”. Conversely, we defined participants with an “improved” clinical outcome as those with “improved” radiological outcome AND asymptomatic at follow-up. Participants who had “intermediate” radiological outcome AND were asymptomatic for TB, were defined as having an “intermediate” clinical outcome (Table 1).

#### 2.3.4. Bacteriological outcomes and developing TB between the TBPS and the follow-up.

The sputum samples of participants who were SE at the follow-up visit were tested using Xpert-Ultra. Participants who had a positive Xpert-Ultra test result were considered to have bacteriologically confirmed MTB and a positive bacteriological outcome. These participants were considered to have developed TB. Participants starting TB treatment in between the TBPS and the follow-up who were not referred for treatment by the MO at the TBPS were also considered to have developed TB since the TBPS.

### 2.4. Statistical analysis

#### 2.4.1. Explanatory variables.

We identified our main explanatory variables as country, and whether the participant reported at the time of the TBPS that that they had previously taken TB treatment or were currently taking TB treatment. Analyses were stratified on these two characteristics, because a previous TB could have led to long-term or permanent lung changes identified on the CXR resulting in a high CAD score irrespective of whether the individual had prevalent TB at the time of the TBPS, as well as potentially causing ongoing TB symptoms [14,15].

#### 2.4.2. Statistical analysis.

All data analysis was conducted using STATA Statistical Software (Stata Corporation Version 15. College Station, TX, USA). A descriptive data analysis was done to describe the sample of individuals who participated in the follow-up survey and compare them with those that, despite being eligible for follow-up, were not followed-up. Among individuals who were followed up, socio-demographic, clinical and behavioural characteristics were compared between those who self-reported previous TB at the TBPS and those who did not, overall and by country. The univariable associations between baseline characteristics (at the time of the TBPS) and “progressed” clinical outcome at follow-up were explored. Significance testing was done using the chi-squared test for categorical variables and the Wilcoxon rank-sum or Kruskal-Wallis test for continuous variables. We fitted logistic regression models for factors associated with “progressed” clinical outcome; adjusted for country, age, sex and self-reporting of previous TB. The characteristics of participants who had positive Xpert-Ultra results at follow-up, and of those that had started and/or completed TB treatment between the TBPS and the follow-up visit, were examined in more detail. For the calculation of the TB incidence rate we included those participants who had positive Xpert-Ultra result at follow-up and those who started TB treatment between the TBPS and the follow-up. We censored those who started TB treatment between the TBPS and the follow-up and we used the treatment initiation date to calculate exposure time. In those in whom we did not have treatment initiation date we used the midpoint date between the TBPS and the follow-up.

## 3. Results

### 3.1. Study flow

**Fig 2:**
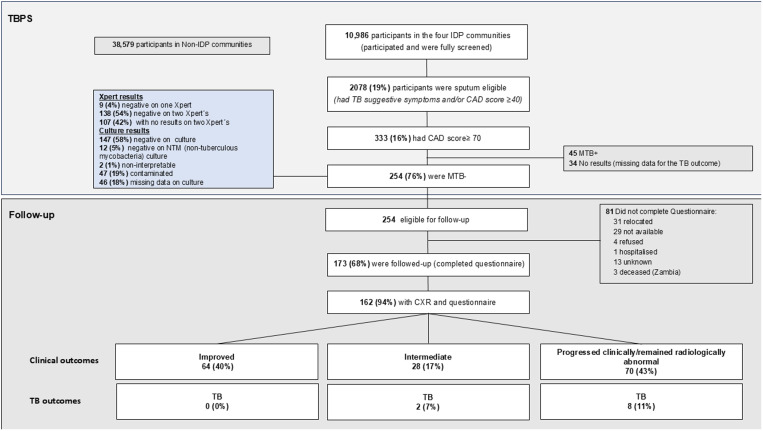
Flow chart showing participant flow from the TB Prevalence Survey to the follow-up.

From 10,986 participants who participated in the TBPS in the four IDP communities 2078 (19%) were sputum eligible (SE). From SE participants 333/2078 (16%) had a CAD score ≥70, out of whom 254 (76%) had bacteriologically unconfirmed TB and were eligible for follow-up. Out of these 254 participants, 244 were classified as Xpert-Ultra negative based on two negative Xpert-Ultra results, and 10 based on one Xpert-Ultra negative result for MTB; 147/254 (58%) of them also had a sputum sample collected for culture testing and the test result was culture-negative (Fig 2).

Amongst the 254 participants who were eligible for follow-up, 173 (68%) were located and consented and 162 (94%) completed the follow-up questionnaire and had a CAD score. Overall, 81/254 (32%) participants, were not followed up due to various reasons, mainly because they were not found at their households after several attempts and/or because they had relocated outside the community (Fig 2). Three participants deceased in Zambia between the TBPS and the follow-up. Participation in the follow-up was lower in SA than in Zambia with no other substantial differences between those who participated in the follow-up and those who did not (S1 Table)

The median time between the TBPS and the follow-up was 9 months (IQR 7–10) overall, 8 months (IQR 7–10) in Zambia and 17 months (IQR 16–18) in SA (S2 Table).

### 3.2. Baseline characteristics

Among the 162 participants who were followed-up, 105 (65%) self-reported previous TB (at the time of the TBPS). Most participants were from Zambia (79%, 128/162,) and male (60%, 97/162). The median age was 44 years [(IQR 36–55] and 24% (39/162) were PLHIV. Those self-reporting previous TB were more likely to be from SA (26% vs 12%), male (67% vs 47%), older (median age 46 vs 40 years), PLHIV (33% vs 7%), reported higher alcohol intake (24% vs 9% drinking twice or more in a month, and current smokers (23% vs 16%) compared to those who did not self-report previous TB (Table 2).

**Table 2:** Characteristics of participants at the time of the TBPS (baseline), initiation of TB treatment and ART between the TBPS and the FU and Xpert testing and results at follow-up, overall and by previous TB^1^.

Variable		Overall N = 162		SR^2^ TB history N = 105		No SR^2^ TB history N = 57		p-value
		n	%	n	%	n	%	
**At the time of the TBPS**								
**Country**	**SA**	34	21.0	27	25.7	7	12.3	0.045
	**Zambia**	128	79.0	78	74.3	50	87.7	
**Sex**	**Male**	97	59.9	70	66.7	27	47.4	0.017
	**Female**	65	40.1	35	33.3	30	52.6	
**Age (years)**	**Median (IQR**^1^)	162	44 (36 - 55)	105	46 (39 - 55)	57	40 (32 - 55)	0.040
**Age group**	**15-29**	20	12.3	6	5.7	14	24.6	0.002
	**30-44**	62	38.3	42	40.0	20	35.1	
	**45-59**	46	28.4	36	34.3	10	17.5	
	**60+**	34	21.0	21	20.0	13	22.8	
**Education**	**None**	17	10.5	9	8.6	8	14.0	0.470
	**Primary**	61	37.7	42	40.0	19	33.3	
	**Secondary**	84	51.9	54	51.4	30	52.6	
**SR**^2^ **HIV-status**	**Positive** ^3^	39	24.1	35	33.3	4	7.0	<0.001
	**Negative**	111	68.5	65	61.9	46	80.7	
	**Unknown**	12	7.4	5	4.8	7	12.3	
**Smoking status**	**Current smoker**	33	20.4	24	22.9	9	15.8	0.051
	**Past smoker**	23	14.2	19	18.1	4	7.0	
	**Non-smoker**	106	65.4	62	59.1	44	77.2	
**Alcohol intake**	**≥2 per month**	30	18.5	25	23.8	5	8.8	0.004
	**Monthly or less**	35	21.6	27	25.7	8	14.0	
	**Never**	97	59.9	53	50.5	44	77.2	
**CAD Score**	**Median (IQR**^1^)	162	84 (74 - 96)	105	85 (73 - 95)	57	83 (75 - 100)	0.620
**IDP follow-up**								
**Sputum eligible (SE)** ^4^	**Yes**	103	63.6	71	67.6	32	56.1	0.018
	**No**	59	36.4	34	32.4	25	43.9	
**Submitted sputum** *(out of those SE)*	**Yes**	92	89.3	63	88.7	29	90.6	0.204
	**No**	11	10.7	8	11.3	3	9.4	
**Xpert result** *(out of those who submitted sputum)*	**Positive** ^5^	5	5.4	4	6.3	1	3.4	0.568
	**Negative**	87	94.6	59	93.7	28	96.6	
**Period between the TBPS and the follow-up**								
**Started TB treatment**	**Yes**	10	6.2	7	6.7	3	5.3	0.750
	**No**	152	93.8	98	93.3	54	94.7	

^1^ Interquartile range.

^2^ Self-reported.

^3^ 37 out of 39 were on antiretroviral therapy.

^4^ Any participants who had symptoms suggestive of TB or a CAD score ≥70 in the follow-up survey (in South Africa samples were not examined on those who had started treatment since the TBPS).

^5^ One MTB detected Trace, three MTB detected Low, and one MTB detected Medium.

### 3.3. CAD-score changes between TBPS and follow-up

**Fig 3:**
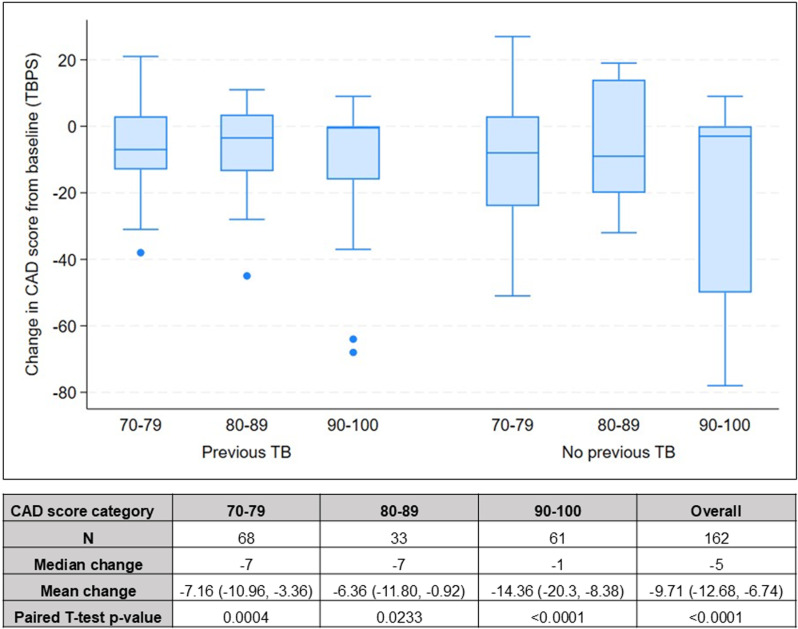
Distribution of change in CAD score by TB history and baseline CAD score category.

Fig 3 shows the distribution of participant’s change in CAD scores by CAD score categories at baseline (at the time of the TBPS), separately for individuals who did or did not self-report previous TB. Overall, there were similar patterns in the change in CAD score in participants with and without previous TB. The median CAD score change among participants with baseline CAD scores of [70–79] and [80–89] was -7, indicating a significant average reduction in CAD score at follow-up. Among those with baseline CAD scores of [70–79] and [80–89], 68% and 61% experienced a reduction in CAD score, respectively. On the other hand, participants with the highest CAD scores [90–100] at TBPS had a median CAD score change of -1, and 48% experienced a reduction in the CAD score.

### 3.4. Clinical outcomes and determinants

Overall, 43.2% (70/162) had “progressed clinically/remained radiologically abnormal”; 17.3% (28/162) had an “intermediate” clinical outcome, and 39.5% (64/162) had an “improved” clinical outcome (Table 3). In the multivariable analysis, those from Zambia and PLHIV were at higher risk of “progressing clinically/remaining radiologically abnormal” (aOR=3.01; 95% CI: 1.23, 7.34 p = 0.015 and aOR=2.78; 95% CI: 1.20, 6.40 p = 0.017 respectively) (Table 3). Disaggregated data of individuals who progressed clinically by symptoms and radiological outcomes are shown in S3 Table.

**Table 3:** Clinical outcomes by baseline characteristics (at the time of the TBPS) and association between baseline characteristics and progressed clinical outcome at follow-up.

Variable		Clinical outcome						Association with progressed clinical outcome			
		Improved		Intermediate		Progressed		Unadjusted OR^4^ (95% CI)	p-value	^*^Adjusted OR^4^ (95% CI	p-value
		n	%	n	%	n	%				
**Overall**		**64**	**39.5**	**28**	**17.3**	**70**	**43.2**				
**Country**	**SA**	13	38.2	12	35.3	9	26.5	Ref	0.03	Ref	0.015
	**Zambia**	51	39.8	16	12.5	61	47.7	2.53 (1.09, 5.84)		3.01 (1.23, 7.34)	
**SR** ^1^ ** TB history**	**Yes**	38	36.2	19	18.1	48	45.7	1.34 (0.69, 2.58)	0.383	1.48 (0.72, 3.02)	0.287
	**No**	26	45.6	9	15.8	22	38.6	Ref		Ref	
**Sex**	**Male**	32	33,0	21	21.6	44	45.4	1.25 (0.66, 2.36)	0.500	1.23 (0.61, 2.48)	0.287
	**Female**	32	49.2	7	10.8	26	40.0	Ref		Ref	
**Age group** **(years)**	**15-29**	11	55.0	1	5.0	8	40.0	Ref	0.992	Ref	0.917
	**30-44**	26	41.9	9	14.5	27	43.5	1.16 (0.41, 3.23)		0.85 (0.28, 2.61)	
	**45-59**	15	32.6	11	23.9	20	43.5	1.15 (0.40, 3.36)		1.14 (0.35, 3.69)	
	**60+**	12	35.3	7	20.6	15	44.1	1.18 (0.39, 3.64)		0.98 (0.29, 3.33)	
**SR** ^1^ ** HIV status**	**Positive** ^2^	11	28.2	4	10.3	24	61.5	2.73 (1.29, 5.79)	0.009	2.78 (1.20, 6.40)	0.017
	**Negative**	46	41.4	24	21.6	41	36.9	Ref		Ref	
**Smoking status**	**Current smoker**	17	51.5	5	15.2	11	33.3	Ref	0.225	Ref	0.431
	**Past smoker**	7	30.4	3	13	13	56.5	1.70 (0.68, 4.21)		1.29 (0.45, 3.68)	
	**Non-smoker**	40	37.7	20	18.8	46	43.4	0.65 (0.29, 1.48)		0.63 (0.25, 1.57)	
**Alcohol intake**	**≥2 per month**	13	43.3	8	26.7	9	30	Ref	0.259	Ref	0.186
	**Monthly or less**	13	37.1	6	17.1	16	45.7	0.97 (0.45, 2.11)		0.81 (0.34, 1.93)	
	**Never**	38	39.2	14	14.4	45	46.4	0.50 (0.21, 1.19)		0.42 (0.16, 1.09)	

^1^Self-reported HIV positive/negative, ^2^37 out of 39 were on antiretroviral therapy, ^3^TB prevalence survey, ^4^Odds ratio.

* Adjusted by country, age, sex and previous TB.

### 3.5. Bacteriological confirmation of TB at follow-up and developing TB from TBPS to follow-up

From the 162 participants that were followed-up 110 (68%) were SE at the follow-up visit (had symptoms suggestive of TB or a CAD score ≥70 at follow-up), 92/110 (84%) provided one sputum sample and five tested positive for MTB on Xpert-Ultra (one MTB detected Trace, three MTB detected Low, and one MTB detected Medium) (Table 2). Out of these five, none had started TB treatment between the TBPS and follow-up and four had “progressed clinically/remained radiologically abnormal” (Table 4).

**Table 4:** Characteristics at the TBPS and follow-up of participants who developed TB.

Variable			Developed TB					
			Xpert + at follow-up (N = 5)		Started TB treatment between TBPS^3^ and follow-up (N = 5)		All (N = 10)	
			n	%	n	%	n	%
**All study participants (N = 162)**			5/162	3	5/162	3	10/162	6
**TBPS** ^3^	**Country**	**Zambia**	4	80	3	60	7	70
		**South Africa**	1	20	2	40	3	30
	**Sex**	**Female**	3	60	1	20	4	40
		**Male**	2	40	4	80	6	60
	**Age group (years)**	**30-44**	0	0	3	60	3	30
		**45-59**	3	60	1	20	4	40
		**60+**	2	40	1	20	3	30
	**SR** ^1^ ** TB history**	**No**	1	20	1	20	2	20
		**Yes**	4	80	4	80	8	80
	**SR** ^1 ^ **HIV status**	**PLHIV** ^2^	1	20	2	40	3	30
		**negative**	4	80	3	60	7	70
	**Symptoms**	**No**	2	40	4	80	6	60
		**Yes**	3	60	1	20	4	40
	**CAD score, median [IQR]**		86 [74 - 93]		83 [76-94]		85 [75-93]	
**Follow-up**	**Symptoms**	No	1	20	3	60	4	40
		**Yes**	4	80	2	40	6	60
	**Clinical progression or remained radiologically abnormal**	**No**	1	20	1	20	2	20
		**Yes**	4	80	4	80	8	80
	**TB treatment between TBPS** ^3^ ** and follow-up**	**No**	5	100	1	20	6	60
		**Yes**	0	0	4	80	4	40
	**Sputum eligible**	**No**	0	0	2	40	2	20
		**Yes**	5	100	3	60	8	80
	**Xpert results**	**positive**	5	100	0	0	5	50
		**negative**	0	0	5	100	5	50
	**CAD score, median [IQR]**		90 [89 – 91]		93 [78-100]		91 [84-97]	

^1^Self-reported, ^2^People living with HIV, ^3^TB Prevalence Survey.

Ten participants started TB treatment between the TBPS and the follow-up. Of these, five were referred for TB treatment by the MO at the TBPS at the TBPS as they were clinically diagnosed with TB. Out of the five who started TB treatment but were not referred by the MO, four had “progressed clinically/remained radiologically abnormal” and three had an Xpert-Ultra result at follow-up and were negative (Table 4).

Ten participants were classified as having developed TB between the TBPS and the follow-up (6%, 10/162) (the 5 participants that had a positive Xpert result at the follow-up and the 5 that started TB treatment between the TBPS and the follow-up who were not referred by the MO at the TBPS) see characteristics in Table 4. The overall TB incidence rate was 7.1% per year (95% CI: 3.8 – 13.3).

## 4. Discussion

Although TBPS’s often find a considerable number of participants with macroscopic pathology in whom the CXR is abnormal but TB is not bacteriologically confirmed, few studies have followed up these individuals, and none has used CAD on CXR for assessing likelihood of TB [4,16]. In this study we found that almost half of participants with high CAD score with bacteriologically unconfirmed TB “progressed clinically/remained radiologically abnormal” over time, with either worsening of CAD scores or CAD score remaining ≥85 or presenting symptoms compatible with active TB. Out of these, 11% had developed TB, while 66% self-reported previous TB in whom post-TB lung disease could likely account for the clinical progression or the remained high CAD scores. The remaining 23% did not report previous TB and had not developed TB by the time of follow-up.

Those who progressed clinically/remained radiologically abnormal were more likely to be from Zambia and living with HIV at the time of the TBPS. HIV is directly associated with TB disease progression, and is also known to cause other respiratory diseases that could manifest with similar symptoms and radiological abnormalities as TB; this could possibly explain the worsening or persistence of symptoms and the high CAD score at follow-up for such individuals [17].

A recent systematic review and metanalysis found that progression from bacteriologically unconfirmed to confirmed TB (based on smear or culture tests) in participants with a baseline CXR suggestive of active tuberculosis (according to a clinician) occurred at an annual rate of 10% (95% CI 6·2–13·3). However, all studies except one were conducted between 1940’s and 1990’s, prior to the HIV epidemic, and most of them were in high-income countries [16]. In a previous TBPS in Cambodia, participants with an “abnormal” CXR (according to clinicians judgement) in whom TB was not confirmed were followed up for two years. They found an incidence rate of bacteriologically confirmed TB of 8.5 per hundred person-year among those participants with a ‘TB-suggestive’ CXR at the TBPS, which is comparable to the rate found in our study (7.1 per hundred person-year). For bacteriological confirmation at follow-up, the Cambodia study used two sputum samples and either smear and/or culture positive results while we used only a one-spot Xpert result, which could have favoured increased TB detection in Cambodia compared to our study. It may be that those participants in our study with bacteriologically confirmed TB at follow-up but who were bacteriologically unconfirmed at the TBPS could have been at early stages of disease at the TBPS, with macroscopic pathology showing at the CXR but still without bacteriological evidence [18]. However, we cannot rule out that those with bacteriologically confirmed TB at follow-up were new infections considering the high transmission rate and the long periods between TBPS and follow-up in some communities. Similarly, we are including those starting TB treatment between the TBPS and the follow-up as having developed TB though we do not know if they were bacteriologically confirmed or not. On the other hand, the proportion of individuals developing TB in the context of a TBPS can be expected to be lower compared to that of programmatic settings due to the higher detection of TB at baseline.

More than half of our follow up study participants self-reported previous TB at the TBPS, which could account for the high CAD score at baseline. In high TB/HIV prevalence settings such as Zambia and South Africa we can expect relatively high proportions of people in the community to have had TB before [19]. Consistent with our findings, a previous study in Zambia reported that people with previous TB were more likely to be male, older, and live with HIV (5). In our study previous TB was also associated with other known TB risk factors such as smoking or alcohol intake [20,21]. Of all those that reported previous TB, 11% developed TB during the follow-up. There is evidence showing a higher risk of incident TB in individuals with previous TB compared with individuals without previous TB [14,15].

The CAD score has been shown to have lower accuracy to detect TB among individuals who had a previous episode of TB, making it difficult to distinguish whether radiological changes are due to previous TB sequelae or a new active TB episode [22]. One study in Zambia found that among presumptive TB patients with prior TB radiographic abnormalities persisted, were common and poorly discriminated between those with and without current active TB [23]. Other studies have shown adverse outcomes in patients with post-tuberculosis lung damage after completion of treatment and high risk of persistently abnormal parenchymal and airway abnormalities seen on imaging and associated respiratory symptoms compared with those without previous TB [22,23].

In this study we found a considerable proportion of participants who had a high CAD score at TBPS in whom clinical progression or the remaining of high CAD scores over time could not be explained by the development of active TB nor by the long-term consequences of previous TB. In the TREATS TBPS, non-TB abnormalities on the CXRs of individuals who had high CAD scores were characterized independently by two radiologists. Pleural effusion/thickening/calcification followed by cardiomegaly were the most prevalent non-TB abnormalities found, some of which could be related to previous TB [24]. In a TBPSs conducted in Malawi and Kenya the most prevalent finding in individuals with abnormal CXRs was also cardiomegaly [7,25]. In Kenya cardiac and pulmonary diseases accounted for 66% of non-TB abnormalities [7]. Further, other studies have consistently shown that digital CXRs detect other abnormalities which might indicate the presence not only of TB but also other communicable and/or non-communicable diseases such as chronic respiratory diseases and cardiovascular disease [26].

This study had several limitations. Firstly, the duration between the TBPS and the follow-up visit was longer than what was initially planned in both sites and longer in SA compared to Zambia. This was a consequence of the interruption of field activities due to the COVID-19 pandemic. Longer time between TBPS and follow-up could predispose to re-infection rather than progression of existing bacteriologically unconfirmed TB. However, longer time could also favour the resolution of TB in those who took treatment between the TBPS and follow-up. Secondly, a considerable proportion (32%) of participants could not be followed up, mostly because they were not found at home after several attempts. This might have led to overestimation of the proportion of those who had “progressed clinically/remained radiologically abnormal”, assuming that those who were not found at home or who relocated elsewhere may have been “healthier”. Conversely, it could have resulted in underestimation, as we did not have information on how many among those not found had died between the TBPS and follow-up in SA, and whether these had TB. Thirdly, in this study, previous TB may have been underreported due to stigma. Fourthly, the use of a high CAD score of 70 to define sputum eligibility at follow up may have limited our ability to capture all participants developing TB over time/since the TBPS and underestimated those who “progressed clinically/remained radiologically abnormal”. There is no established threshold for CAD score and WHO recommends adapting the cut-off depending on the objective of testing, the number of available bacteriological tests, and the underlying TB prevalence [27]. Another limitation may be that diagnosis of bacteriological TB at follow-up relied on a single-spot sputum which could result in underestimating the number of participants progressing to TB over time [10]. Conversely, we could have overestimated this number by including one Xpert trace result as a positive result [28,29].

## 5. Conclusion

With new WHO guidelines recommending digital CXR and automated image interpretation for systematic screening in high-prevalence populations a considerable proportion of patients with a high CAD score without bacteriologically confirmed TB will be identified. Many of these patients may have had a past TB episode or other conditions resulting in pulmonary lesions identified in the CXR leading to clinical progression and/or to persistence of high CAD scores, which may need to be investigated. Also, some of these participants may be at risk of progressing to TB over time and following them up may be worth considering as part of case finding activities. New approaches are needed to support the management of this group.

## Supporting information

S1 Table Characteristics of TBPS participants with abnormal chest-X-ray but without proven microbiological TB who participated in the IDP follow-up compared to those who did not. (DOCX)

S2 Table Distribution time (in months) between the TBPS and the follow-up by country. (DOCX)

S3 Table Participants who progressed clinically by symptoms and radiological outcomes at follow-up. (DOCX)
