# Supplementary material for: Clinical outcomes of participants of a TB prevalence survey with an abnormal chest X-ray but no evidence of TB disease after a median follow-up of 9 months in Zambia and South Africa
Source: PLOS Glob Public Health. 2025 Jun 20;5(6):e0003787. doi: 10.1371/journal.pgph.0003787 (PMC12180716; doi:10.1371/journal.pgph.0003787)
Supplement: S1 Table — (DOCX) [file pgph.0003787.s001.docx]

**S1 Table Characteristics of TBPS participants with abnormal chest-X-ray but without proven microbiological TB who participated in the IDP follow-up compared to those who did not**

| **Variable** | **Total** | **Participated** | **Not participated** | **p-value** |
| --- | --- | --- | --- | --- |
|  | **N=254** | **N=162** | **N=92** |  |
| **Country** |  |  |  | <0.001 |
| SA | 84 (33.1%) | 34 (21.0%) | 50 (54.3%) |  |
| Zambia | 170 (66.9%) | 128 (79.0%) | 42 (45.7%) |  |
| **Sex** |  |  |  | 0.880 |
| Male | 153 (60.2%) | 97 (59.9%) | 56 (60.9%) |  |
| Female | 101 (39.8%) | 65 (40.1%) | 36 (39.1%) |  |
| **Age, median (IQR)** | 43.0 (34.0-55.0) | 44.0 (36.0-55.0) | 40.0 (33.0-53.0) | 0.057 |
| **Age group** |  |  |  | 0.240 |
| 15-24 | 17 ( 6.7%) | 9 ( 5.6%) | 8 ( 8.7%) |  |
| 25-34 | 53 (20.9%) | 28 (17.3%) | 25 (27.2%) |  |
| Primary | 83 (32.7%) | 61 (37.7%) | 22 (23.9%) |  |
| Secondary/Higher | 146 (57.5%) | 84 (51.9%) | 62 (67.4%) |  |
| **Ever been treated for TB** |  |  |  | 0.810 |
| Yes | 166 (65.4%) | 105 (64.8%) | 61 (66.3%) |  |
| No | 88 (34.6%) | 57 (35.2%) | 31 (33.7%) |  |
| **Self-reported HIV status** |  |  |  | 0.870 |
| Positive | 59 (23.2%) | 39 (24.1%) | 20 (21.7%) |  |
| Negative | 175 (68.9%) | 111 (68.5%) | 64 (69.6%) |  |
| Unknown | 20 ( 7.9%) | 12 ( 7.4%) | 8 ( 8.7%) |  |
| **ART if HIV positive** |  |  |  | 0.300 |
| On ART | 57 (97%) | 37 (95%) | 20 (100%) |  |
| Not on ART | 2 ( 3%) | 2 ( 5%) | 0 ( 0%) |  |
| **Smoking status** |  |  |  | 0.008 |
| Non-smoker | 155 (61.0%) | 106 (65.4%) | 49 (53.3%) |  |
| Past smoker | 31 (12.2%) | 23 (14.2%) | 8 ( 8.7%) |  |
| Current smoker | 68 (26.8%) | 33 (20.4%) | 35 (38.0%) |  |
| **Alcohol intake** |  |  |  | 0.590 |
| Never | 148 (58.3%) | 97 (59.9%) | 51 (55.4%) |  |
| Monthly or less | 54 (21.3%) | 35 (21.6%) | 19 (20.7%) |  |
| At least twice in a month | 52 (20.5%) | 30 (18.5%) | 22 (23.9%) |  |
| **CAD Score, mean (sd)** | 85.3 (10.9) | 84.7 (11.2) | 86.5 (10.4) | 0.190 |
