# Supplementary figures and images for: Clinical outcomes of participants of a TB prevalence survey with an abnormal chest X-ray but no evidence of TB disease after a median follow-up of 9 months in Zambia and South Africa
Source: PLOS Glob Public Health. 2025 Jun 20;5(6):e0003787. doi: 10.1371/journal.pgph.0003787 (PMC12180716; doi:10.1371/journal.pgph.0003787)

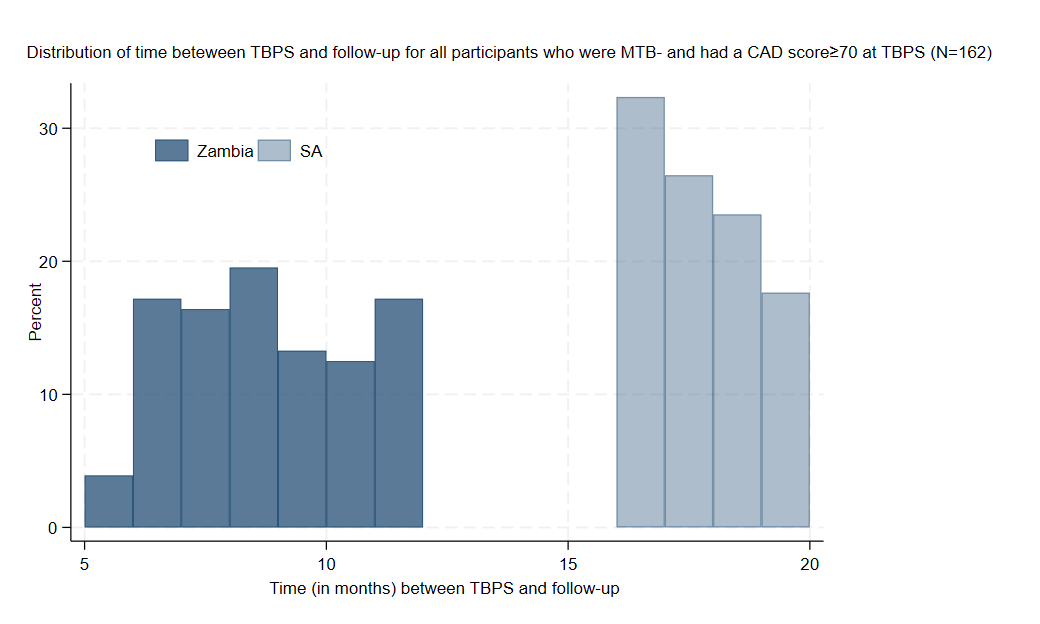

Supplement: S2 Table — (DOCX) [file pgph.0003787.s002.docx]
