# Supplementary material for: Clinical outcomes of participants of a TB prevalence survey with an abnormal chest X-ray but no evidence of TB disease after a median follow-up of 9 months in Zambia and South Africa
Source: PLOS Glob Public Health. 2025 Jun 20;5(6):e0003787. doi: 10.1371/journal.pgph.0003787 (PMC12180716; doi:10.1371/journal.pgph.0003787)
Supplement: S3 Table — (DOCX) [file pgph.0003787.s003.docx]

| **Variable** | | **Clinical Progression** | | | | | | |
| --- | --- | --- | --- | --- | --- | --- | --- | --- |
|  |  | **Symptoms only** | | | **Radiological progression only** | | **Both symptoms and radiologically** | |
|  |  | **n** | **%** | **n** | | **%** | **n** | **%** |
| **Overall** | | **8** | **11,4** | **44** | | **62,9** | **18** | **25,7** |
| **Country** | **SA** | 0 | 0.0 | 8 | | 88.9 | 1 | 11.1 |
|  | **Zambia** | 8 | 13.1 | 36 | | 59.0 | 17 | 27.9 |
| **TB history** | **Yes** | 4 | 8.3 | 31 | | 64.6 | 13 | 27.1 |
|  | **No** | 4 | 18.2 | 13 | | 59.1 | 5 | 22.7 |
| **Sex** | **Male** | 5 | 11.4 | 29 | | 65.9 | 10 | 22.7 |
|  | **Female** | 3 | 11.5 | 15 | | 57.7 | 8 | 30.8 |
| **Age group**  **(years)** | **15-29** | 1 | 12.5 | 7 | | 87.5 | 0 | 0.0 |
|  | **30-44** | 4 | 14.8 | 17 | | 63.0 | 6 | 22.2 |
|  | **45-59** | 2 | 10.0 | 12 | | 60.0 | 6 | 30.0 |
|  | **60+** | 1 | 6.7 | 8 | | 53.3 | 6 | 40.0 |
| **SR^1^ HIV status** | **Positive** | 4 | 16.7 | 15 | | 62.5 | 5 | 20.8 |
|  | **Negative** | 4 | 9.8 | 25 | | 61.0 | 12 | 29.3 |
| **HIV on ART** | **On ART** | 4 | 17.4 | 14 | | 60.9 | 5 | 21.7 |
|  | **No ART** | 0 | 0.0 | 1 | | 100.0 | 0 | 0.0 |
| **HIV status and ART^2^** | **SR^1^ HIV+, Not on ART^2^** | 0 | 0.0 | 1 | | 100.0 | 0 | 0.0 |
|  | **SR^1^ HIV+, on ART^2^** | 4 | 17.4 | 14 | | 60.9 | 5 | 21.7 |
|  | **Tested HIV+** | 0 | 0.0 | 3 | | 75.0 | 1 | 25.0 |
|  | **Tested HIV-** | 1 | 4.8 | 14 | | 66.7 | 6 | 28.6 |
|  | **Not tested** | 3 | 14.3 | 12 | | 57.1 | 6 | 28.6 |
| **Smoking status** | **Current smoker** | 1 | 9.1 | 7 | | 63.6 | 3 | 27.3 |
|  | **Past smoker** | 2 | 15.4 | 9 | | 69.2 | 2 | 15.4 |
|  | **Non-smoker** | 5 | 10.9 | 28 | | 60.9 | 13 | 28.3 |
| **Alcohol intake** | **≥2 in a month** | 0 | 0.0 | 7 | | 77.8 | 2 | 22.2 |
|  | **Monthly or less** | 3 | 18.8 | 10 | | 62.5 | 3 | 18.8 |
|  | **Never** | 5 | 11.1 | 27 | | 60.0 | 13 | 28.9 |

**S3 Table Participants who progressed clinically by symptoms and radiological outcomes at follow-up**
